# Clinical and Epidemiological Profile of Patients with Sinonasal Papilloma in a University Hospital *

**DOI:** 10.1055/s-0045-1811638

**Published:** 2026-03-03

**Authors:** Sanderson Endrew Gomes Leão, Michelle Queiroz Aguiar Brasil, João Coêlho Neto, Carolina Cincurá Barreto, Marcus Miranda Lessa, Clara Mônica Figueredo de Lima

**Affiliations:** 1 Department of Otorhinolaryngology, Hospital Universitário Professor Edgard Santos, Universidade Federal da Bahia, Salvador, BA, Brazil; 2 School of Medicine, Universidade Federal da Bahia, Salvador, BA, Brazil; 3 Department of Otorhinolaryngology, Universidade Federal do Sul da Bahia, Teixeira de Freitas, BA, Brazil

**Keywords:** sinonasal papilloma, nasal endoscopic surgery, rhinology

## Abstract

**Introduction:**

Sinonasal papilloma is the most common benign tumor of the nasal cavity, with the inverted subtype being the most frequent. Its etiology remains highly controversial, but *Human Papillomavirus* (HPV) is believed to be associated with its pathogenesis. In addition to being locally aggressive, this tumor is characterized by high recurrence rates and malignant transformation potential.

**Objective:**

To describe the clinical and epidemiological profile of patients diagnosed with sinonasal papilloma who underwent surgery at a University Hospital.

**Methods:**

A cross-sectional study was performed analyzing the profile of patients diagnosed with sinonasal papilloma who underwent surgical treatment between January 1, 2021, and December 31, 2023. Variables such as sociodemographic, symptoms, smoking history, tumor location, Krouse staging, surgical techniques, histological subtypes, recurrence, and malignant transformation were analyzed.

**Results:**

A higher prevalence was observed among women (52.4%), with a mean age of 54.2 ± 12 years old, and without a history of smoking (61.9%). Nasal obstruction was the most reported symptom, with the left nasal cavity being more frequently affected. Most cases were classified as Krouse stage T3, and 85.7% of all analyzed cases were managed exclusively through endoscopic approaches. Inverted papilloma was the most common subtype (66.7%). Only 9.5% and 4.8% of patients experienced recurrence and malignant transformation, respectively.

**Conclusion:**

Sinonasal papilloma was more prevalent in middle-aged women with no history of smoking. The diagnosis and treatment of sinonasal papilloma remain delayed due to nonspecific symptoms and limited access to specialized healthcare in Brazil. Long-term follow-up is crucial for monitoring potential recurrence and malignant transformation.

## Introduction

Sinonasal papilloma is the most common benign tumor of the nasal cavity, accounting for ∼ 0.4 to 4.7% of all nasal and paranasal sinus neoplasms. Its embryological origin lies in the ectoderm of the olfactory placode, which subsequently differentiates into the primitive nasal sac and ultimately forms the nasal mucosa (also known as Schneiderian epithelium). 1

According to the World Health Organization (WHO), it can be divided into three different subtypes: inverted (or transitional/Ringertz cells), exophytic (or everted/vestibular), and oncocytic (or columnar cells). 1 2

The oncocytic subtype is the least frequent, representing ∼ 3 to 6% of cases. 3 Histologically, it is characterized by inverted and exophytic epithelial growth with numerous intraepithelial microcysts and abundant eosinophilic cytoplasm. 4 Unlike other subtypes, its pathogenesis does not involve *Human Papillomavirus* (HPV) infection. However, clinically, these pathologies are very similar and can also progress toward malignancy, with invasive squamous cell carcinoma being the most reported tumor. 5

Exophytic papilloma, on the other hand, is the second most common subtype, with a prevalence ranging from 18 to 50%, according to the medical literature. 3 Its histology is typically fungiform and squamous. Epistaxis tends to be the most common symptom, as the tumor generally originates from the nasal septum, unlike other subtypes that typically arise from the paranasal sinuses (mainly maxillary). 6 Furthermore, recurrence rates are lower, and malignancy is rare. 1

Inverted papilloma (IP) is the most common subtype, with a higher prevalence in males (4 to 5 times more than in females) starting in the 4 ^th^ decade of life. 7 It exhibits three main characteristics: relative local aggressiveness, high recurrence rates, and potential association with carcinoma. 8 Histologically, there is a neoplastic invagination of the epithelium into the underlying stroma. 1

The etiology of inverted papilloma remains controversial. Numerous studies have been conducted on this topic, but their findings often conflict. Exposure to HPV and *Epstein-Barr* virus (EBV) has been suggested to be associated with its development. *Human papillomavirus* (HPV) disrupts cell cycle regulation and growth, targeting the tumor suppressor p53, which, along with viral integration, may induce oncogenesis, serving as a marker for neoplastic transformation. Furthermore, HPV subtypes 16 and 18 appear to be associated with higher rates of malignant transformation. Smoking also seems to play a role in papilloma malignancy and recurrence rates. 9

Clinically, patients present with a range of nasal complaints, the most common being unilateral nasal obstruction, rhinorrhea, epistaxis, facial pain, and hyposmia. Due to its nonspecific nature, diagnosis is often delayed. Other possible symptoms include anosmia, epiphora, facial paresthesia, nasal speech, and proptosis (in cases involving the lamina papyracea). A classic triad, present in ∼ 40 to 60% of advanced cases, includes facial asymmetry, a palpable/visible tumor in the oral cavity, and a visible intranasal tumor. 10

Inverted papilloma is typically diagnosed at later stages, between 1 to 4 years after the onset of symptoms. Approximately 4 to 23% of cases are diagnosed incidentally in asymptomatic patients. On physical examination and nasal endoscopy, the tumor appears lobulated, gray-reddish, firmer than an inflammatory polyp, and characteristically “raspberry-like.” Upon palpation, it is friable and prone to bleeding. 8 Definitive diagnosis is established through biopsy, although some cases may yield false negatives due to their resemblance to benign polyps. 10

Imaging studies are crucial for diagnosis and surgical planning. 10 Computed tomography (CT) is nonspecific, often showing a unilateral, homogeneous, isodense lesion, typically located in the middle nasal meatus, associated with microcalcifications and bone erosion. Focal hyperostosis may indicate the tumor's implantation site. Magnetic resonance imaging (MRI) complements CT and may reveal signs of malignancy. 8

In 2000, Krouse developed a staging system for sinonasal papilloma based on radiological tumor extension. 11 Although it has not proven superior to others, it is the most widely used today due to its simplicity, facilitating comparisons across studies. 8 A recent systematic review showed that Krouse stage T3 inverted papillomas have an increased recurrence risk of 51% compared with T2 lesions. 12

The treatment for IP is surgical in all cases. 1 Until the mid-1990s, external approaches were commonly used. However, with technological advancements, endoscopic nasal surgery has become the gold standard for treating IP. Nevertheless, endoscopic surgery is indicated only for tumors with limited extension. Tumors involving the frontal sinus; lateral, inferior, or anterior walls of the maxillary sinus; with extrasinus involvement; or carcinoma associated with papilloma require external or combined techniques. Radiotherapy is also an option in cases of carcinoma association or surgical infeasibility. 8

The present study aims to describe the clinical and epidemiological profile of patients diagnosed with sinonasal papilloma who underwent surgery at a University Hospital, to analyze the surgical techniques used for papilloma removal, and to evaluate recurrence and malignancy rates.

## Methods

### Study Design

A cross-sectional study based on medical records analysis.

### Population and Setting

The study population consisted of patients of both genders, ≥ 18 years old, diagnosed with sinonasal papilloma, who were treated at the Otorhinolaryngology Outpatient Clinic of the Professor Edgard Santos University Hospital. All patients underwent surgical treatment between January 1, 2021, and December 31, 2023.

The sample size was determined based on the number of patients operated on during this period, following the proposed inclusion and exclusion criteria.

### Study Variables

The analyzed variables included sociodemographic data, such as gender and age. Clinical symptoms, smoking history, tumor location, Krouse staging, surgical technique performed, histological subtype, recurrence, and malignant transformation were also assessed.

### Inclusion Criteria

- Patients of both genders, ≥18 years old;
- Diagnosed with sinonasal papilloma, confirmed through pathological examination;
- Patients who underwent surgical treatment at the referenced hospital from January 2021 onwards.

### Exclusion Criteria

- Patients < 18 years old;
- Patients with a history of sinonasal papilloma previously treated surgically;
- Medical records with incomplete data (e.g., missing surgical description, CT scan report, or pathological results).

### Data Analysis

All collected data were stored on electronic devices. The data was tabulated in Excel (Microsoft) software and used to create charts and tables.

Data analysis was performed using SPSS Statistics for Mac (IBM Corp.) 24.0. Numerical variables were assessed for normality through graphical analysis and the Shapiro-Wilk test, presented as mean and standard deviation (SD) for normally distributed data, or as median and interquartile range for non-normal distributions. Categorical variables were reported as absolute numbers and percentages. The independent Student's T-test or the Mann-Whitney test and the Chi-squared or the Fisher's exact test were used for bivariate analysis of categorical variables. A *p* -value < 0.05 was considered statistically significant.

### Ethical Aspects

The present study, involving human subjects, was registered on the Plataforma Brasil and was submitted to the Research Ethics Committee of the Professor Edgard Santos University Hospital under approval number 6.816.834.

## Results

From January 2021 to December 2023, 21 patients ( Table 1 ) were diagnosed with sinonasal papilloma at the University Hospital Professor Edgard Santos. A higher prevalence was observed among women (52.4%) with a mean age of 54.2 ± 12 years old (range: 32–74 years old). When asked about smoking history, 38.1% of the patients reported having smoked at some point in their lives.

**Table 1:** Clinical characteristics of papilloma cases treated at a specialized service

Variables	*n* = 21
**Age in years** , mean ± SD	54.2 ± 12
**Female gender** , *n* (%)	11 (52.4)
**Smoking history** , *n* (%)	8 (38.1)
**Symptom duration in months** , median (p25–p75)	35.0 (22.0–60.5)
**Presence of nasal obstruction** , *n* (%)	19 (90.5)
**Presence of rhinorrhea** , *n* (%)	18 (85.7)
**Presence of headache** , *n* (%)	10 (47.6)
**Presence of olfactory alteration** , *n* (%)	11 (52.4)
**Presence of epistaxis** , *n* (%)	12 (57.1)
**Tumor location** , *n* (%) Right nasal cavity (RNC) Left nasal cavity (LNC) Bilateral	5 (23.8) 15 (71.4) 1 (4.8)
**Krouse CT staging** , *n* (%) T1 T2 T3 T4	3 (14.3) 3 (14.3) 14 (66.7) 1 (4.8)
**Histopathological findings** , *n* (%) Inverted Exophytic Oncocytic Combined	14 (66.7) 2 (9.5) 3 (14.3) 2 (9.5)
**Presence of neoplasia** , *n* (%)	1 (4.8)
**Surgical procedure** , *n* (%) Endoscopic surgery Combined endoscopic + Caldwell-Luc	18 (85.7) 3 (14.3)
**Disease recurrence** , *n* (%)	2 (9.5)

Abbreviation: SD, standard deviation.

Regarding clinical symptoms, nasal obstruction (unilateral or bilateral) was the most frequent complaint, reported by 90.5% of the patients, followed by rhinorrhea, epistaxis, olfactory dysfunction, and headache. Patients had a median symptom duration of 35 months (interquartile range: p25–75). Tumors were predominantly located in the left nasal cavity in 71.4% of cases, with only 1 patient presenting bilateral lesions. The most common Krouse staging was T3 (tumor involving the lateral and/or inferior portion of the maxilla or the frontal and/or sphenoid sinus), accounting for 66.7% of the cases.

Most surgical procedures (85.7%) were performed using exclusive endoscopic techniques. Three cases required combined external access (Caldwell-Luc). No open surgeries were performed.

Histopathological examinations revealed that IP was the most prevalent subtype, present in 66.7% of the samples, followed by oncocytic and exophytic subtypes, found in 14.3% and 9.5% of the cases, respectively. Two patients had combined histopathological findings. The first presented with inverted and exophytic papilloma, while the second had exophytic papilloma with in situ squamous cell carcinoma. The latter was the only malignant transformation case in the sample during the study period.

Recurrence occurred in two cases. The first was a 56-year-old male patient with no history of smoking, presenting all evaluated symptoms, with a lesion in the right nasal cavity classified as Krouse T3. Histopathology showed a combination of inverted and exophytic papillomas, and the surgical approach involved combined techniques (endoscopic nasal surgery and Caldwell-Luc). The interval between symptom onset and the first surgery was ∼ 38 months. Recurrence was identified ∼ 15 months after the initial procedure. The second recurrence case was a 32-year-old male patient with a smoking history, presenting only nasal obstruction and epistaxis, classified as Krouse T4. The patient had bilateral lesions, with in situ squamous cell carcinoma and exophytic papilloma in the right nasal cavity and in situ squamous cell carcinoma in the left nasal cavity. The time between symptom onset and the first surgery was ∼ 57 months, with recurrence identified 17 months later.

Bivariate analysis ( Table 2 ) comparing clinical characteristics between cases with or without recurrence showed statistically significant differences ( *p* ≤ 0.05) for tumor location ( *p* = 0.003) and histopathological findings ( *p* = 0.000). Approximately 80% of lesions originating from the left nasal cavity did not recur, nor did cases of IP, which accounted for 66.7% of the sample. Both recurrence cases presented combined histopathological findings in biopsy results.

**Table 2:** Comparison of clinical characteristics between papilloma cases with and without disease recurrence

Variables	Recurrence		
	Yes *n* = 2	No *n* = 19	*p-value*
**Age in years** , mean ± SD	44.0 ± 17.0	55.3 ± 11.5	0.213*
**Female gender** , *n* (%)	0 (0.0)	11 (57.9)	0.214**
**Smoking history** , *n* (%)	1 (50.0)	7 (36.8)	1.00**
**Symptom duration in months** , median (p25–p75)	47.5 (38.0-57.0)	29 (20.0–64.0)	0.214***
**Presence of nasal obstruction** , *n* (%)	2 (100.0)	17 (89.5)	1.00**
**Presence of rhinorrhea** , *n* (%)	1 (50.0)	17 (89.5)	0.271**
**Presence of headache** , *n* (%)	1 (50.0)	9 (47.4)	1.00**
**Presence of olfactory alteration** , *n* (%)	1 (50.0)	10 (52.6)	1.00**
**Presence of epistaxis** , *n* (%)	2 (100.0)	10 (52.6)	0.486**
**Tumor location** , *n* (%) Right nasal cavity (RNC) Left nasal cavity (LNC) Bilateral	1 (50.0) 0 (0.0) 1 (50.0)	4 (21.1) 15 (78.9) 0 (0.0)	0.003**
**Krouse CT staging** , *n* (%) T1 T2 T3 T4	0 (0.0) 0 (0.0) 1 (50.0) 1 (50.0)	3 (15.8) 3 (15.8) 13 (68.4) 0 (0.0)	0.17**
**Histopathological findings** , *n* (%) Inverted Exophytic Oncocytic Combined	0 (0.0) 0 (0.0) 0 (0.0) 2 (100.0)	14 (66.7) 2 (9.5) 3 (14.3) 0 (0.0)	0.000**
**Presence of neoplasia** , *n* (%)	1 (50.0)	0 (0.0)	0.095**
**Combined endoscopic + Caldwell-Luc surgery** , *n* (%)	1 (50.0)	2 (10.5)	0.271**

**Abbreviations** : CT computed tomography; SD, standard deviation.

**Notes** : * Student's t-test for age.

** Pearson's Chi-squared or Fisher's exact test for categorical variables.

*** Mann-Whitney test for symptom duration.

## Discussion

Sinonasal papilloma is a benign tumor that typically occurs during the 5 ^th^ or 6 ^th^ decades of life, 13 with a higher prevalence in males. 14 In the present study, most patients were women with no history of smoking, with a mean age of 54.2 years old. However, five patients were excluded due to prior nasal papilloma surgery (an exclusion criterion), all of whom were male. This may explain the predominance of women in the sample. Additionally, smoking has not been shown to be a risk factor for the development of the disease, although it has been associated with recurrence rates. 15 16

Regarding symptomatology, nasal obstruction and rhinorrhea were the most frequent complaints, present in 90.5% and 85.7% of cases, respectively, with the left nasal cavity being the most affected side. These findings align with results from similar studies. 7 17 The extended duration of symptoms (from onset to surgery) and the fact that most cases were already classified as Krouse T3 (tumor involving the lateral and/or inferior maxilla or the frontal and/or sphenoid sinuses) reflect delayed diagnoses, likely due to limited access to specialized healthcare in Brazil's unified health system (SUS, *Sistema Único de Saúde* , in Portuguese). Consequently, delayed treatment initiation directly impacts patients' quality of life.

Approximately 85% of surgeries utilized exclusively endoscopic techniques, reflecting a global trend toward this approach, which has shown comparable outcomes to external access techniques while reducing morbidity and avoiding scarring. Nonetheless, external approaches remain necessary in complex cases, such as papillomas with challenging endoscopic access or extrasinus invasion. 18 19 It is also noteworthy that the hospital where the present study was conducted is a referral center for rhinology in the state of Bahia, Brazil, staffed by professionals with extensive experience in nasal endoscopic surgery.

As expected, the sample was predominantly composed of IPs, which is consistent with findings from various studies. 17 18 20 21 Regarding malignancy risk, papillomas can exhibit varying degrees of histological differentiation, from atypia and dysplasia to situ carcinoma or even squamous cell carcinoma. Malignancy may occur synchronously with the papilloma or metachronously after its resection. 22 During the study period, only one patient exhibited malignant transformation. This patient had bilateral lesions, with in situ squamous cell carcinoma and exophytic papilloma on the right side. This finding is unusual, as malignancy typically arises in inverted and oncocytic papillomas. 23 A Japanese case report also described exophytic papilloma evolving into squamous carcinoma, emphasizing the rarity of such cases and the limited literature available on this subject. 24

Regarding recurrences, two cases were identified. The first involved a 56-year-old male patient with a combined histopathological subtype (inverted and exophytic papillomas). A U.S. study conducted at a tertiary center reviewed sinonasal papilloma cases over 15 years, revealing that patients with combined histopathological subtypes had higher recurrence rates and more frequent complaints of epistaxis (consistent with our participant's findings). 6 Additionally, Krouse T3 classification 12 and younger age 25 appear to be associated with disease recurrence. Younger age may relate to the increased frequency of more aggressive tumors in this population.

The second case of recurrence involved the patient with malignant transformation (male, 32 years old, with a smoking history and bilateral lesions). Bilaterality, combined with smoking habits and age, already constitute risk factors for lesion recurrence. However, studies have shown that the origin site of recurrent lesions often coincides with the primary tumor's location. This suggests a potential association with incomplete removal of the initial lesion. 26 Therefore, prolonged follow-up is necessary to monitor both potential recurrence and the risk of carcinoma development. 27 Annual endoscopic follow-up is recommended, supplemented with imaging studies when pathological changes are observed during nasoendoscopy. 7

The bivariate analysis in the present study demonstrated a statistically significant relationship between lesion location and histological subtype, whether associated with recurrence or not. It was noted that most cases of inverted papilloma, as well as those originating from the left nasal cavity, did not exhibit disease recurrence. However, no data in the literature suggests that lesion laterality impacts disease recurrence. Moreover, contrary to the findings of the present study, inverted papilloma is a subtype associated with high recurrence rates. 8

The present study's limitations include the small sample size, which restricted the statistical power of the analyses, and the short follow-up period, which limited assessments of malignancy and recurrence rates.

## Conclusion

Sinonasal papilloma is a benign but locally aggressive tumor with risks of malignant transformation and recurrence. Limited access to healthcare services in Brazil contributes to delays in diagnosing and treating affected patients. At a university hospital in Salvador, state of Bahia, Brazil, during the analyzed period, papilloma was more prevalent among middle-aged women, most of whom were nonsmokers. The primary clinical complaint was nasal obstruction. Nasal endoscopic surgery was the most frequently employed therapeutic method, with combined approaches reserved for more advanced and complex cases. Inverted papilloma was the most common histological subtype. Recurrence and malignant transformation rates were 9.5% and 4.8%, respectively. Long-term outpatient follow-up is recommended to enable early detection of potential recurrence and malignancy. Extending the study with a longer patient follow-up period could provide more robust and statistically significant data.
